# Supplementary material for: Somatic gene delivery faithfully recapitulates a molecular spectrum of high-risk sarcomas
Source: Nat Commun. 2025 Jun 16;16:5283. doi: 10.1038/s41467-025-60519-5 (PMC12170903; doi:10.1038/s41467-025-60519-5)
Supplement: Supplementary file 1 — Supplementary Information [file 41467_2025_60519_MOESM1_ESM.pdf]

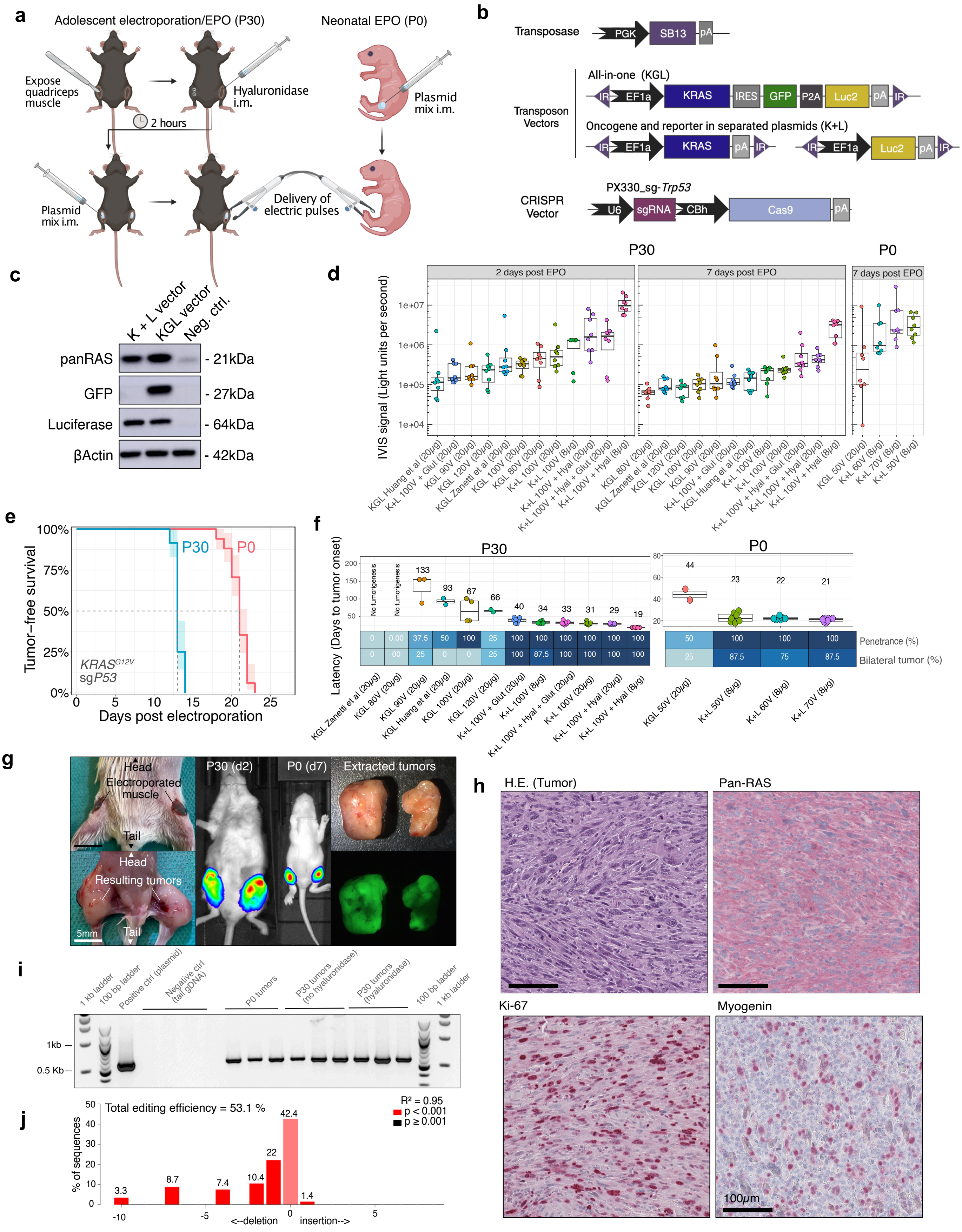

Supplementary Figure 1. An optimized protocol allows efficient *in vivo* genetic manipulation of mouse muscle tissue. a) Scheme of electroporation procedure in P30 and P0 animals. **b)** Illustration of utilized transposon and CRISPR vectors. KGL = EF1a-KRAS-IRES-eGFP-P2A-Luc; K+L = EF1a-KRAS + PGK-Luc. Created in BioRender. Banito, A. (2025) https://BioRender.com/immsfj7. **c)** Western blot of HEK293T cells transfected with vectors used for electroporation. **d)** Quantification of IVIS signal at day 2 and 7 after electroporation as a surrogate for muscle transfection efficiency in P30 and P0 CD1 mice (IVIS at day 2 was not feasible in P0 group). Electroporation settings labelled Huang et al (Huang et al., 2017) and Zanetti et al (Zaneti et al., 2019) al were obtained from literature. n=4 mice electroporated bilaterally. **e)** Kaplan-Meier curves of tumor-free survival after electroporation (K+L) using the ideal optimized conditions. **f)** Boxplots of tumor-free survival in CD1 mice ordered by median from high to low with attached heatmaps of penetrance and laterality of tumor onset in P30 and P0 animals. Mean plotted on top of boxplot. **g)** Exemplary images of mice during the electroporation procedure, upon removal of tumors and bioluminescence imaging. Scale bars equal 5mm. **h)** H&E histographs exemplifying typical histological appearance of tumors driven by oncogenic *KRAS* and *Trp53* inactivation. Scale bars equal 100µm. **i)** Agarose gel electrophoresis from tumor gDNA for transposed *RAS.* **j)** TIDE analysis knockout efficiency in tumor compared to wildtype (tail) control tissue. Boxplots display individual values, median, interquartile range (IQR). Whiskers extend to the most extreme data points within 1.5 times the IQR from the lower and upper quartiles. Source data are provided as a Source data file.

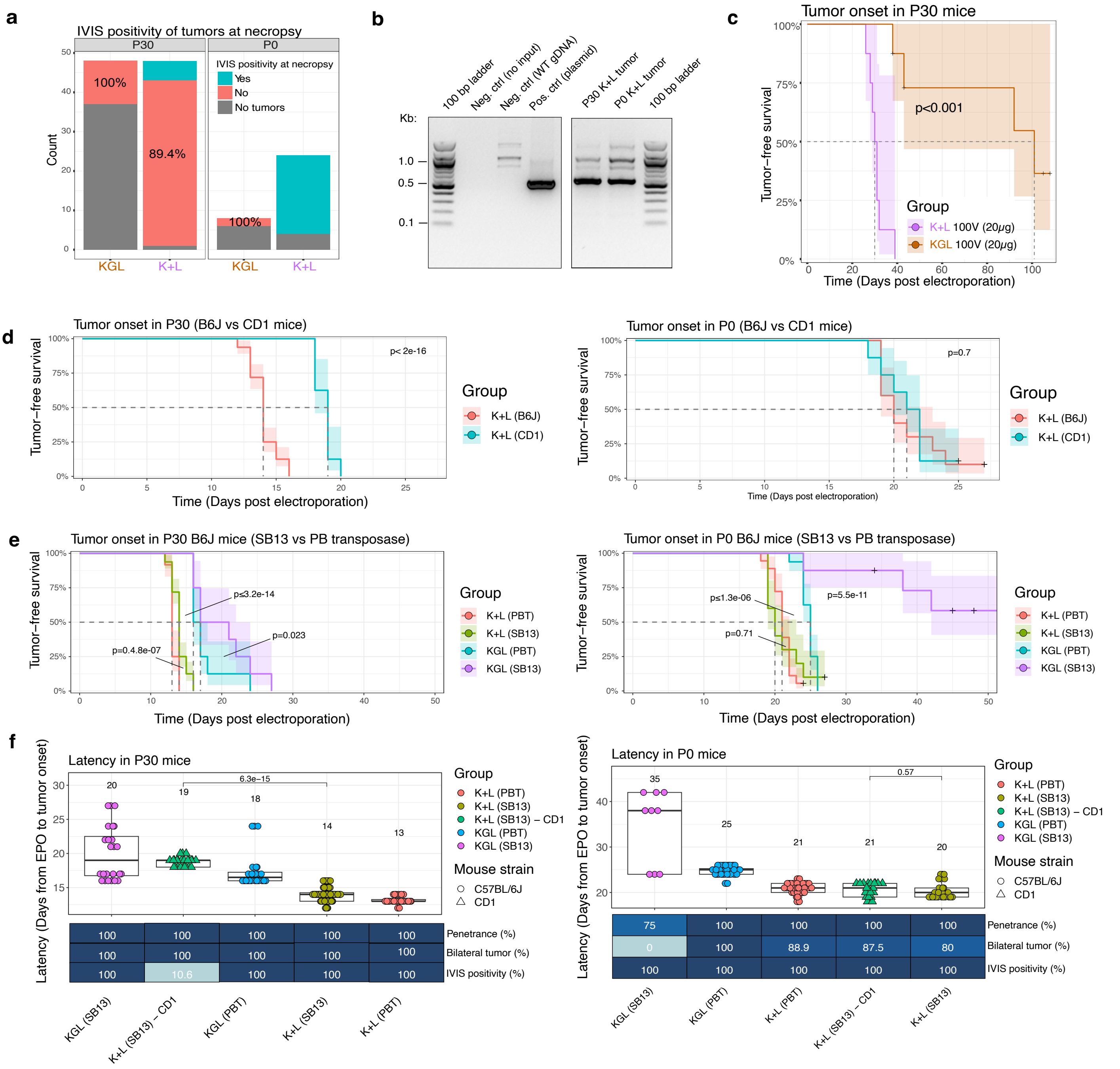

Supplementary Figure 2. Optimization of transfection efficiency and tumor take in CD1 and C57BL/6J mice. a) IVIS positivity of tumors in CD1 mice before necropsy. **b)** Detection of Luciferase transposon in tumor gDNA by PCR. **c)** Tumor-free survival between K+L and KGL tumors in CD1 mice. **d)** Tumor-free survival using K+L vectors in B6J compared to CD1 mice upon P30 and P0 EPO. **e)** Tumor-free survival in B6J mice using K+L vs KGL vectors and SB13 vs PBT transposase upon P30 and P0 EPO. **f)** Boxplots of tumor-free survival in B6J and CD1 mice using the ideal electroporation procedure as determined in panels in Extended Data Figure 1d, ordered by median from high to low with attached heatmaps of penetrance, laterality of tumor onset and IVIS positivity before necropsy in P30 and P0 animals. Boxplots display individual values, median, interquartile range (IQR). Whiskers extend to the most extreme data points within 1.5 times the IQR from the lower and upper quartiles.Source data are provided as a Source data file.

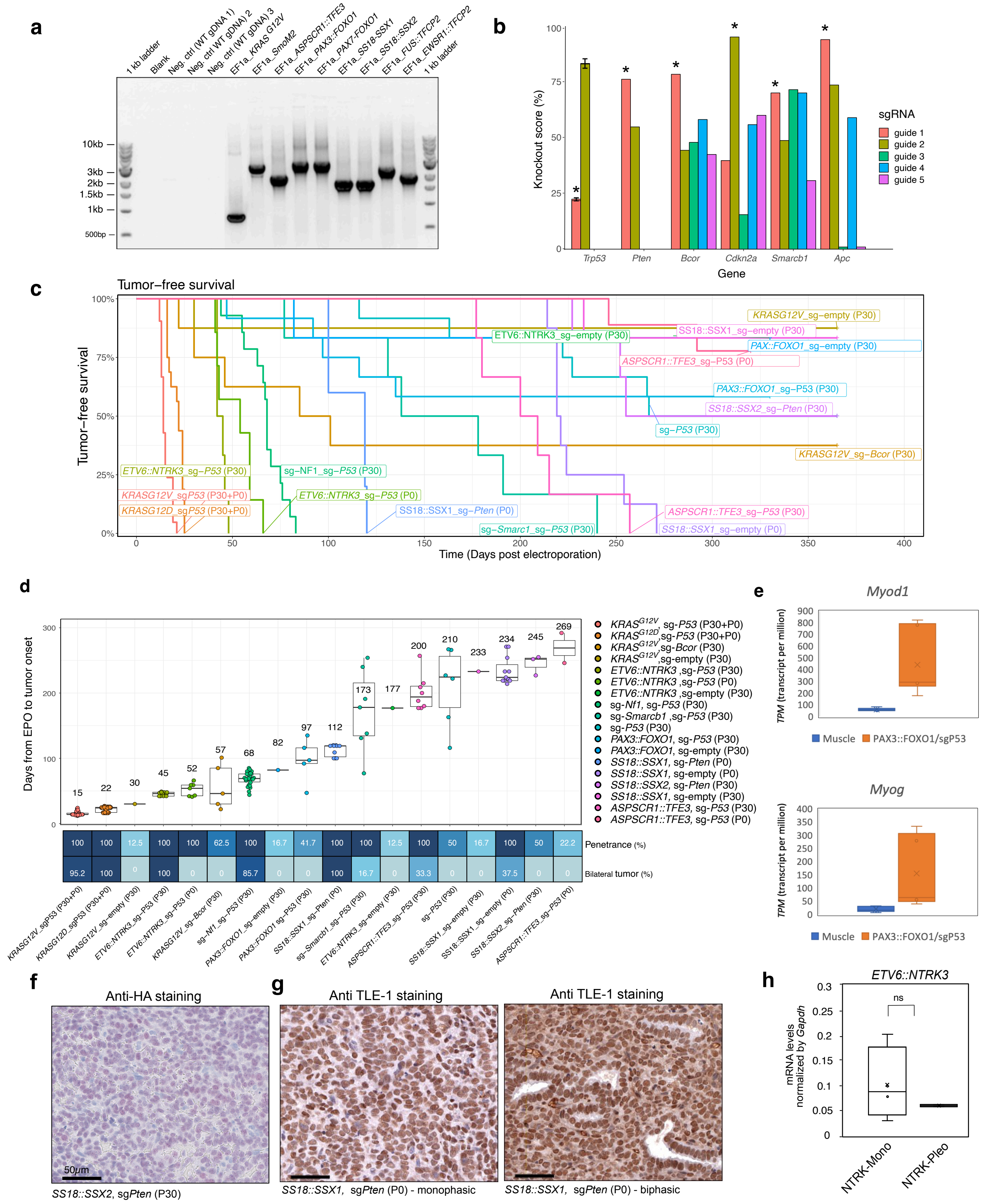

Supplementary Figure 3. Efficiency of sarcoma induction in mice. a) Exemplary genotyping PCRs of transposon vectors used to induce tumors *in vivo*. **b)** Knockout scores quantified using TIDE analysis in N2A cells (murine neuroblastoma line), three days after transient transfection with PX330 vectors carrying the respective sgRNAs. *Trp53* guide 1 (Dow et al., 2015) shows a falsely low knockout score due to a base mismatch in the *Trp53* target region in N2A cells. Experiments for *Trp53* were performed with n = 3 (SD depicted) to ensure reproducibility of the assay. **c)** Kaplan-Meier curves of tumor free survival of all genetic combinations that were successful in tumor induction (n=18). **d)** Tumor-free survival across the mouse sarcoma cohort from panel **c)** as boxplots sorted by median onset with attached heatmaps of penetrance and bilateral tumor fraction. Mean plotted on top of boxplot. **e)** Myod1 and Myogenin normalized gene read counds (RNAseq) for normal muscle (n=6) and PAX3::FOXO1 tumors (n=5). **f)** Anti-HA IHC staining of murine tumors driven by *Flag-HA-SS18::SSX2*. **g)** Anti TLE-1 IHC staining of murine SS18::SSX-driven tumors. **h)** Quantitative RT-PCR analysis for *ETV6::NTRK3* in NTRK-Mono (n=4) and NTRK-Pleo (n=2) tumors. Source data are provided as a Source data file.

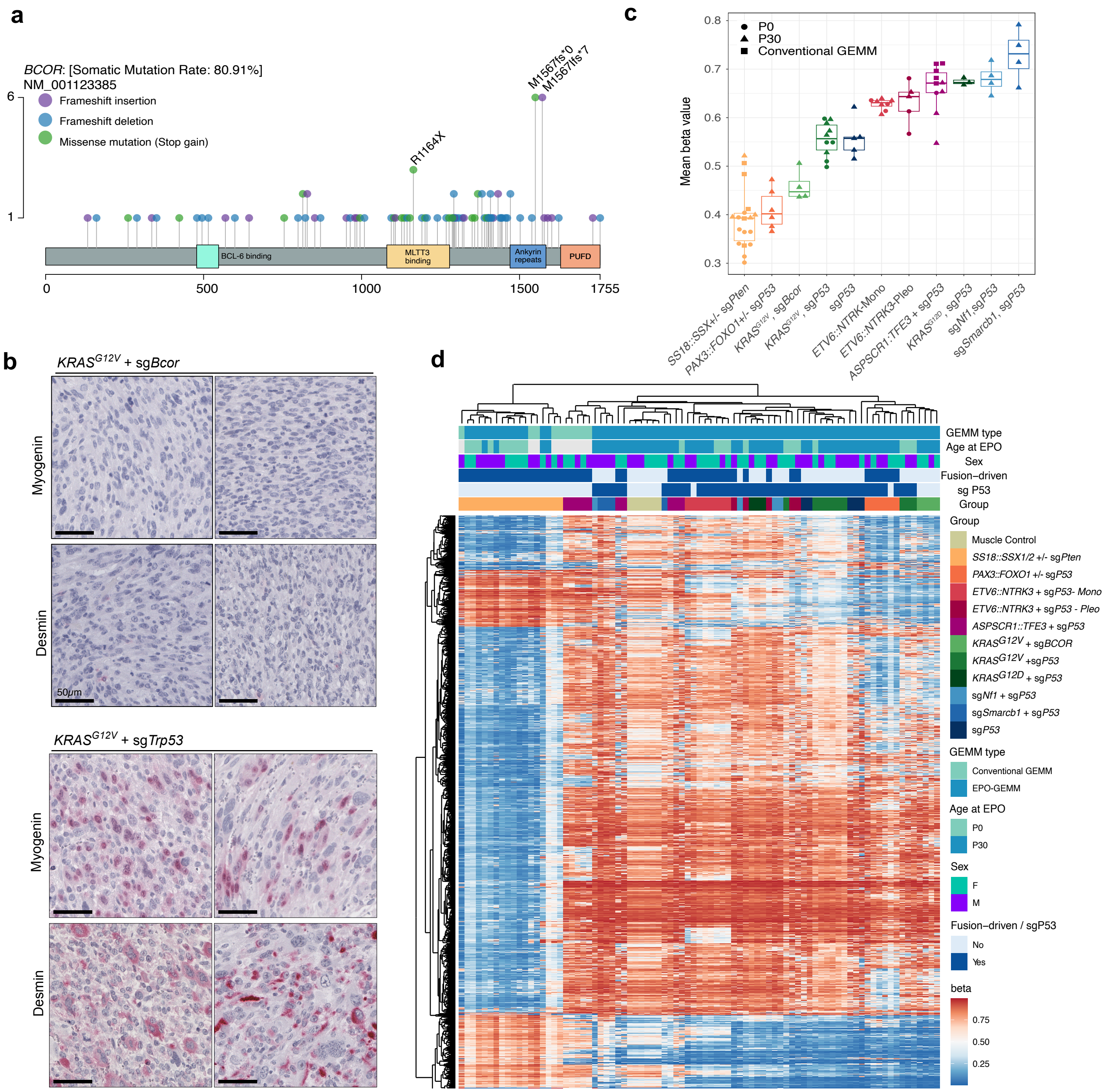

Supplementary Figure 4. *BCOR* inactivation and DNA methylation patterns in mouse sarcomas. a) BCOR mutations identified in human rhabdomyosarcoma, re-analysis of previously published data (Shern et al., 2021). **b)** Exemplary IHC histographs for mesenchymal and muscle-differentiation markers desmin and myogenin, across *KRAS*^{G12V}/sg-*Bcor* and *KRAS*^{G12V}/sg-*Trp53* tumors. **c)** Mean methylation beta values as boxplots ordered by median. Boxplots display individual values, median, interquartile range (IQR). Whiskers extend to the most extreme data points within 1.5 times the IQR from the lower and upper quartiles. **d)** Heatmap view of DNA methylation based on the top 10,000 differentially methylated CpG sites across the mouse sarcoma cohort. n ≥ 3 tumors per group. Source data are provided as a Source data file.

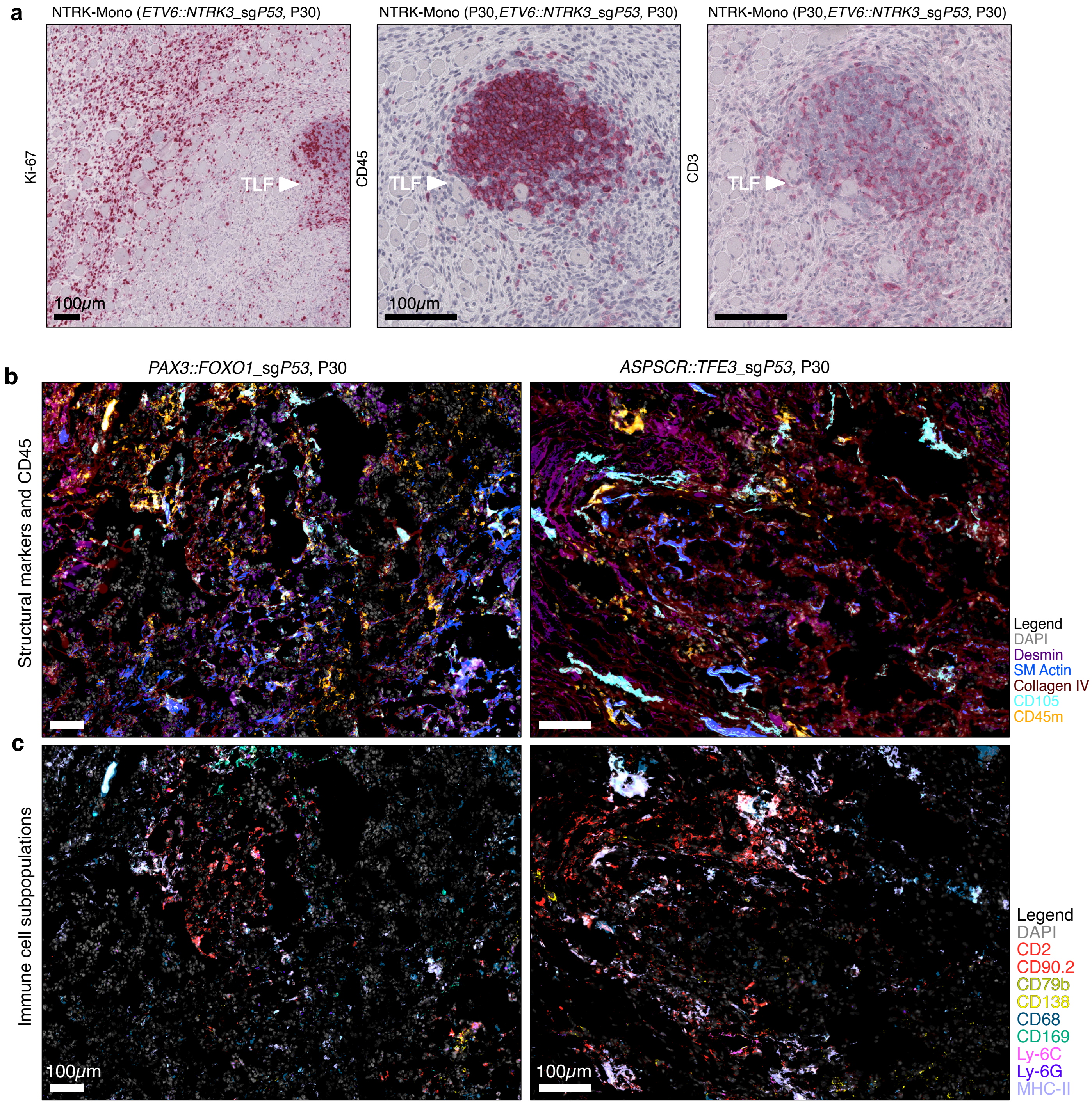

Supplementary Figure 5. Immune infiltration phenotypes of mouse sarcomas. a) Exemplary IHC histographs of immune infiltration in mouse sarcomas. TLF refers to tertiary lymphoid follicles, which were occasionally observed. Matching tumor regions in neighboring slides are depicted as H&E and IHC for Ki-67, CD45, CD3, demonstrating decreased proliferation rate of tumors cells in TLF proximity. **b-c)** Exemplary multiplex IF histographs of mouse sarcomas. Panels in **b)** show structural markers, using DAPI for nuclear staining, mesenchymal marker desmin, cytoskeletal markers smooth muscle actin and collagen IV, endothelial marker CD105 and CD45 for immune cells. Panels in **c)** illustrate immune cell subpopulations in matching regions using DAPI for nuclear staining, CD2 and CD90.2 for T cells, CD79 for B cells, CD138 for plasma cells, CD68 as a pan macrophage marker, CD169 for CD169-positive macrophages, Ly-6G and Ly-6C for granulocytic and monocytic myeloid-derived suppressor cells (g-/m-MDSCs) and MHCII. Scale bars=100µm. Source data are provided as a Source data file.

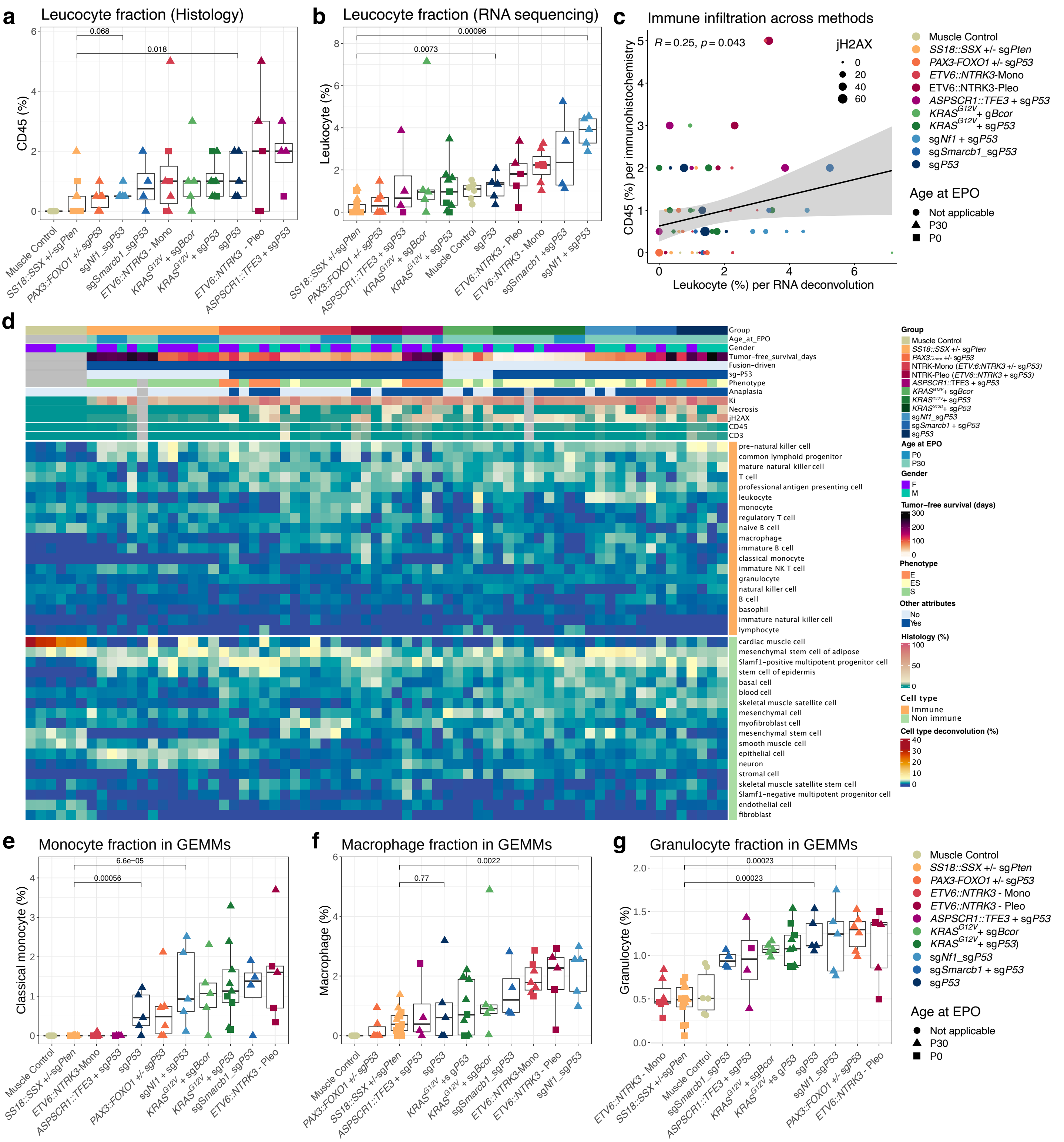

Supplementary Figure 6. Immune cell deconvolution in mouse sarcomas. a-c) Comparison of leukocyte quantification based on IHC (**a**) and cell type deconvolution (**b**) based on RNA sequencing using CibersortX and the publicly available single-cell RNA-seq Tabula muris data set as a reference matrix (Tabula Muris Consortium et al., 2018). Panel **c)** shows a Pearson correlation between the two methods. **d)** Heatmap view of immune and non-immune cells using CibersortX and Tabula muris. **e-g)** Monocyte, macrophage and granulocyte fractions from panel **d)** depicted as median-sorted boxplots. P-values of boxplots were determined by unpaired two-sided Wilcoxon tests and corrected for multiple testing by Bonferroni-Holm method. n ≥ 4 tumors per group. Boxplots display individual values, median, interquartile range (IQR). Whiskers extend to the most extreme data points within 1.5 times the IQR from the lower and upper quartiles.Source data are provided as a Source data file.

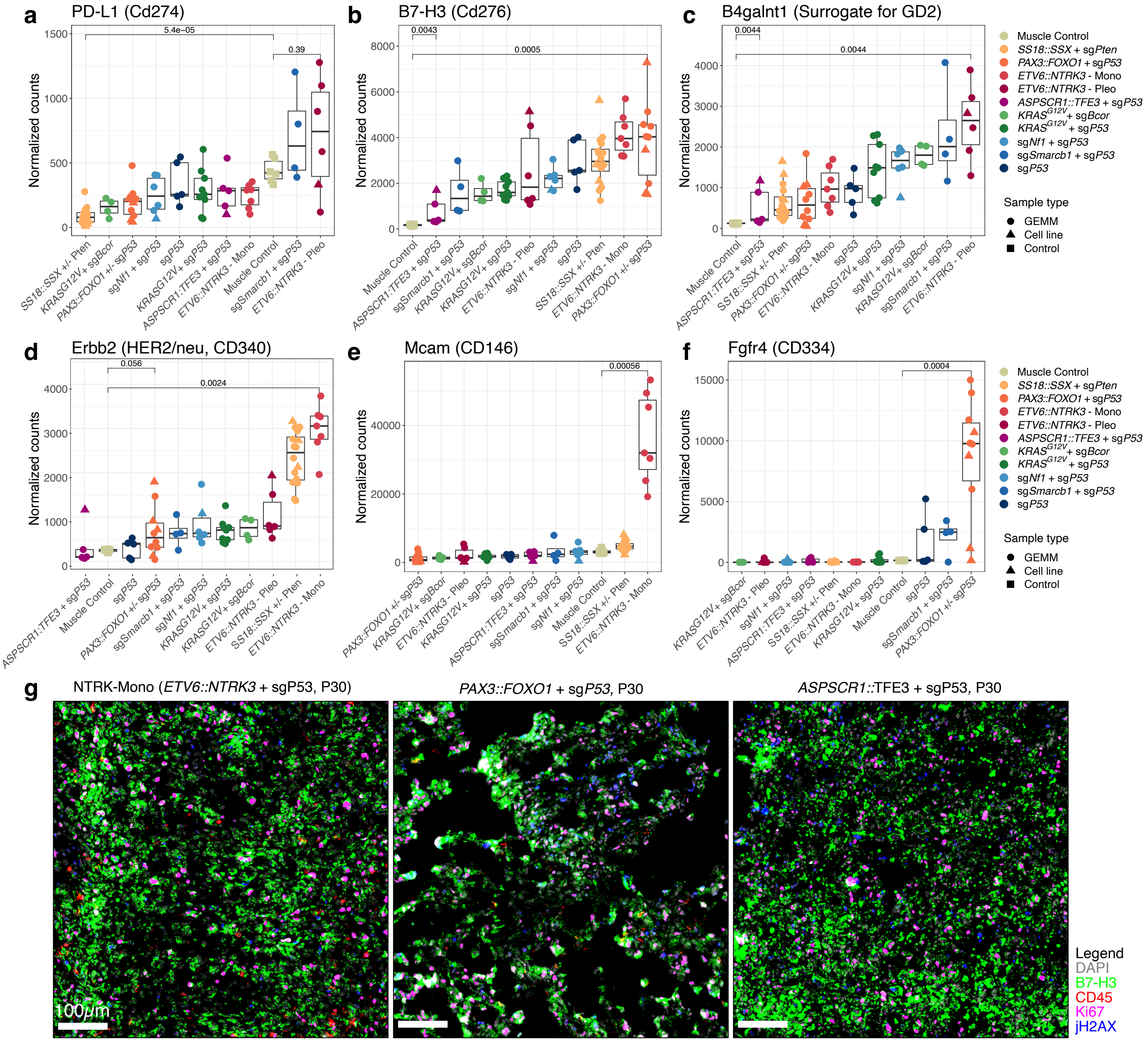

Supplementary Figure 7. Expression of immunotherapy targets in mouse sarcomas. a-f) Median-ordered boxplots, depicting normalized immunotherapy target gene expression across mouse sarcomas based on RNA-sequencing. The chosen pan-cancer and entity-specific targets, were previously identified in human cancer cohorts. For Ganglioside GD2, expression of producing enzyme B4galnt1 was plotted, previously established as a highly correlative marker gene (Sorokin et al., 2020). n ≥ 4 tumors per group. **g)** Exemplary multiplexed IF histographs depicting B7-H3 expression amidst DAPI for nuclear staining, CD45 for immune cells, jH2AX for DNA doubles strand breaks and proliferation markers Ki-67. Scale bars equal 100µm. Boxplots display individual values, median, interquartile range (IQR). Whiskers extend to the most extreme data points within 1.5 times the IQR from the lower and upper quartiles. P-values of boxplots were determined by unpaired two-sided Wilcoxon tests and corrected for multiple testing by Bonferroni-Holm method.Source data are provided as a Source data file.

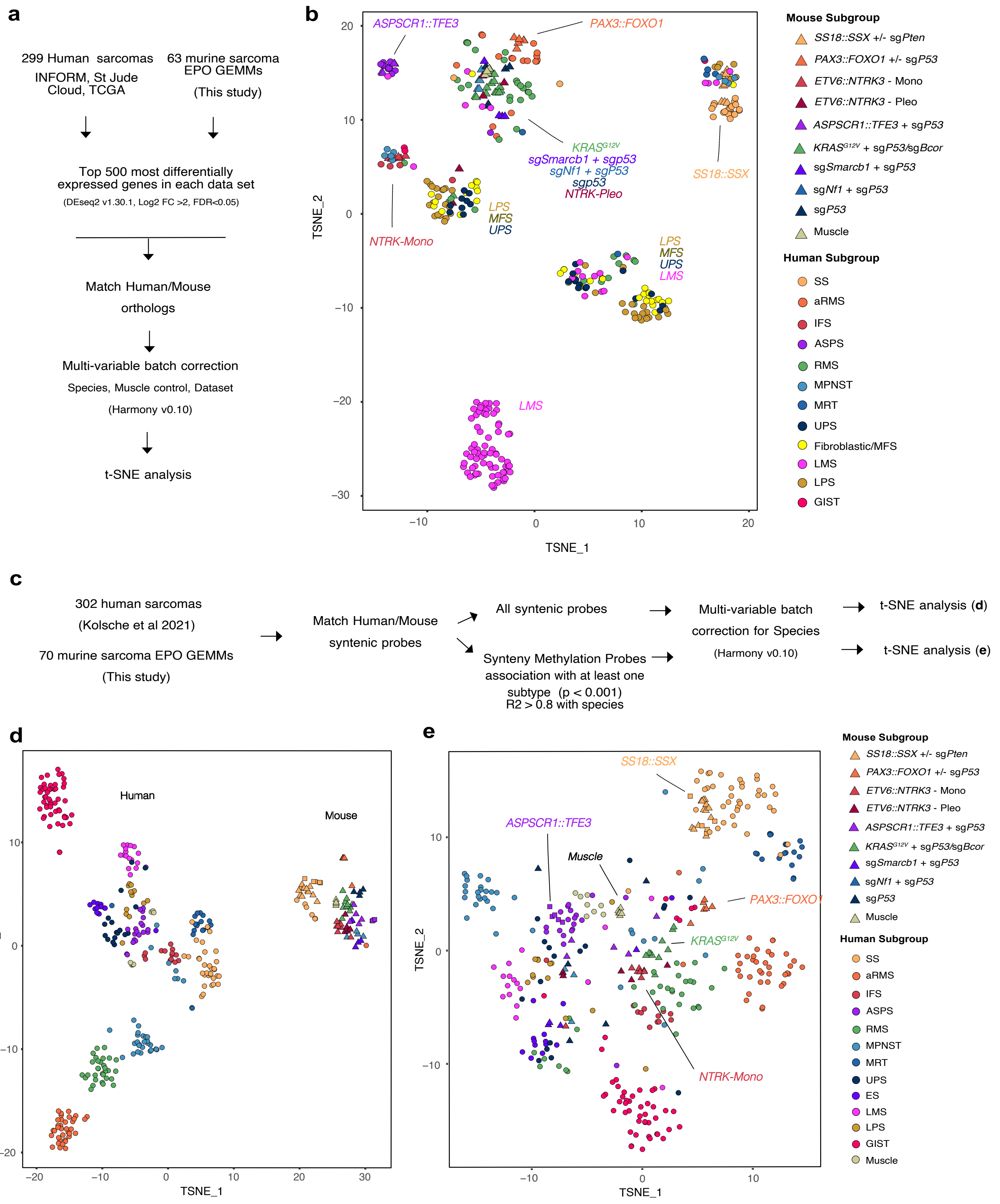

Supplementary Figure 8. Murine sarcomas resemble a spectrum of human sarcomas. a) Analysis scheme of cross-species sarcoma analysis based on RNA sequencing. **b)** tSNE clustering based on cross-species transcriptome analysis of mouse (n=63) and human (n=299) sarcoma specimens. **c)** Analysis scheme of cross-species sarcoma analysis based on DNA methylation **d, e)** tSNE clustering based on M values for all 15218 syntenic probes (d) or 38 selected probes (e) for mouse (n=70) and human (n=302) sarcoma specimens. (SS) Synovial Sarcoma, (aRMS) Alveolar Sarcoma, (IFS) Infantile Fibrosarcoma, (ASPS) Alveolar Soft Part Sarcoma, (RMS) Rhabdomyosarcoma, (MPNST) Malignant Peripheral Nerve Sheath Tumor, (MRT) Malignant Rhabdoid Tumor, (UPS) Undifferentiated Pleomorphic Sarcoma, (ES) Epithelioid sarcoma, (MFS) Myofibroblastic Tumor, (LMS) Leiomyosarcoma, (LPS) Liposarcoma , (GIST) Gastrointestinal stromal Tumor. Source data are provided as a Source data file.

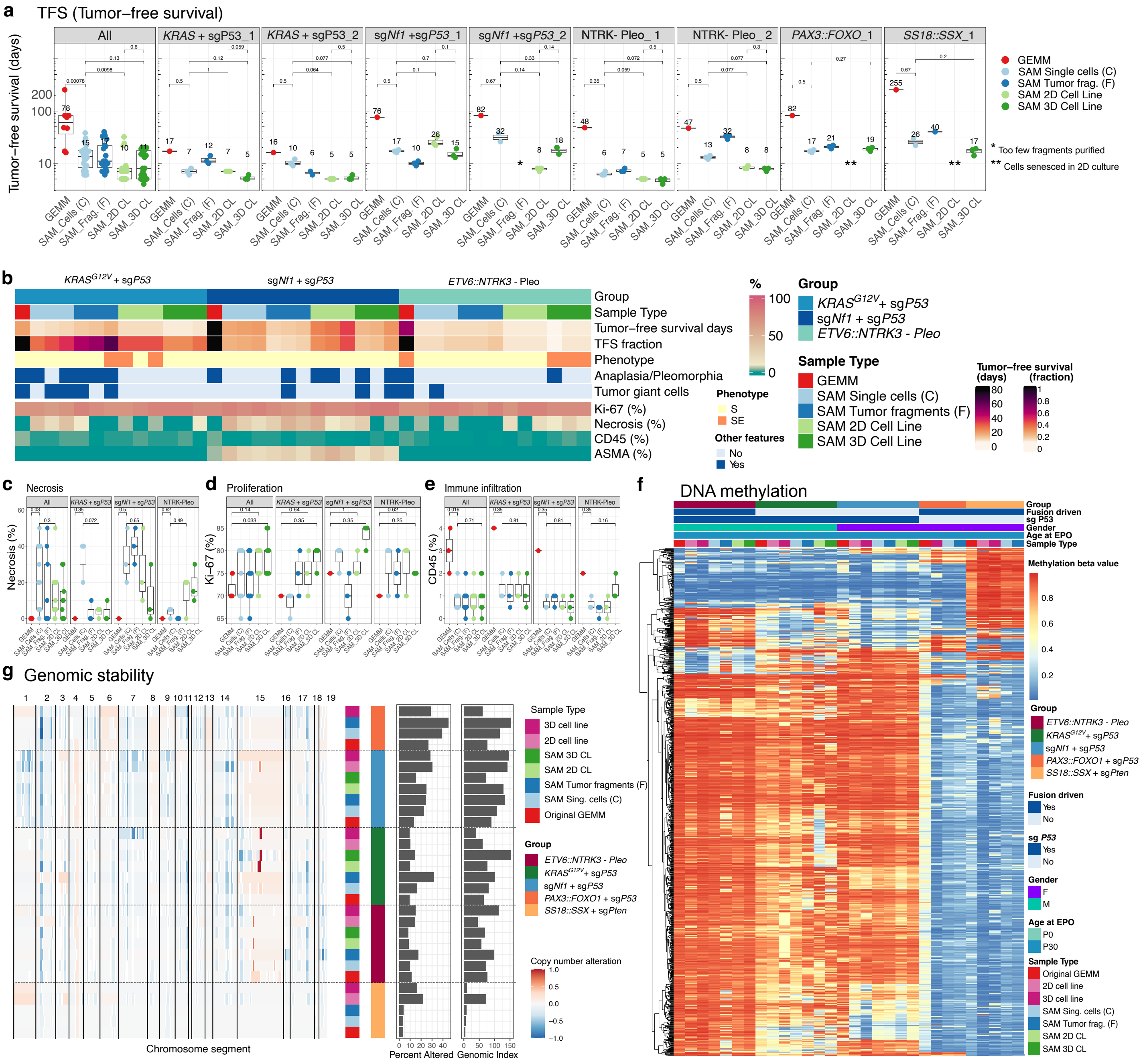

Supplementary Figure 9. Syngeneic allograft models faithfully recapitulate GEMMs. a) Tumor-free survival of SAMs compared to corresponding GEMMs. **b)** Heatmap view of mouse sarcomas of three different entities, systematically compared across original GEMM and different engraftment types, quantified for six morphological and IHC features by blinded expert pathology review. Asymmetric color scale for combined visualization of low (CD45) and high-scoring antigens. **c-e)** Histological features (Necrosis, Ki-67 and CD45 score) from panel **b)** visualized as boxplots. Boxplots display individual values, median, interquartile range (IQR). Whiskers extend to the most extreme data points within 1.5 times the IQR from the lower and upper quartiles. P-values of boxplots were determined by unpaired two-sided Wilcoxon tests and corrected for multiple testing by Bonferroni-Holm method. n = 3 tumors per engraftment group. **f)** Heatmap view of DNA methylation based on the top 10,000 differentially methylated CpG sites comparing GEMMs and corresponding SAMs. n=1 tumor per group. **g)** Genomic stability in SAMs compared to GEMMs, visualized as copy number variations (CNV) profiles derived from DNA methylation data, condensed as Percent altered (≤/≥ .1) and Genomic Index. n=1 tumor per group. Source data are provided as a Source data file.
