# Supplementary material for: Somatic gene delivery faithfully recapitulates a molecular spectrum of high-risk sarcomas
Source: Nat Commun. 2025 Jun 16;16:5283. doi: 10.1038/s41467-025-60519-5 (PMC12170903; doi:10.1038/s41467-025-60519-5)
Supplement: Supplementary file 2 — Description of Additional Supplementary Files [file 41467_2025_60519_MOESM2_ESM.pdf]

Description of Additional Supplementary Files

Supplementary Data 1. Muscle electroporation literature. Table summarizing conditions previous used in muscle electroporation studies. Relative to Supplementary Figure 1.

Supplementary Data 2. List of genes in k-means clusters. List of genes in each k-mean cluster and corresponding gene ontology analysis. Relative to Figure 4c.

Supplementary Data 3. Genes and probes for mouse-human comparisons. Genes and DNA methylation probes used for mouse-human comparisons. Relative to Figure 5 and supplementary Figure 8.

Supplementary Data 4. Plasmids used in this study. Lists of plasmids used in this study and their origin.

Supplementary Data 5. sgRNAs and Tide oligos. Lists of sgRNA and tide oligos and corresponding sequences.

Supplementary Data 6. List of Antibodies. Lists of antibodies used in this study including dilution used and application.

Supplementary Data 7. Mouse strains. Information about the mouse strains used in this study.

Supplementary Data 8. Genotyping primers. Lists of genotyping primers and corresponding sequences.
