# Supplementary material for: Somatic gene delivery faithfully recapitulates a molecular spectrum of high-risk sarcomas
Source: Nat Commun. 2025 Jun 16;16:5283. doi: 10.1038/s41467-025-60519-5 (PMC12170903; doi:10.1038/s41467-025-60519-5)
Supplement: Supplementary file 11 — Reporting Summary [file 41467_2025_60519_MOESM11_ESM.pdf]

nature portfolio

Corresponding author(s): Ana Banito, Robert Autry

Last updated by author(s): Apr 17, 2025

# Reporting Summary

Nature Portfolio wishes to improve the reproducibility of the work that we publish. This form provides structure for consistency and transparency in reporting. For further information on Nature Portfolio policies, see our Editorial Policies and the Editorial Policy Checklist.

## Statistics

For all statistical analyses, confirm that the following items are present in the figure legend, table legend, main text, or Methods section.

| n/a | Confirmed | |
|---|---|---|
| ☐ | ☒ | The exact sample size (*n*) for each experimental group/condition, given as a discrete number and unit of measurement |
| ☐ | ☒ | A statement on whether measurements were taken from distinct samples or whether the same sample was measured repeatedly |
| ☐ | ☒ | The statistical test(s) used AND whether they are one- or two-sided<br>*Only common tests should be described solely by name; describe more complex techniques in the Methods section.* |
| ☒ | ☐ | A description of all covariates tested |
| ☐ | ☒ | A description of any assumptions or corrections, such as tests of normality and adjustment for multiple comparisons |
| ☐ | ☒ | A full description of the statistical parameters including central tendency (e.g. means) or other basic estimates (e.g. regression coefficient) AND variation (e.g. standard deviation) or associated estimates of uncertainty (e.g. confidence intervals) |
| ☐ | ☒ | For null hypothesis testing, the test statistic (e.g. *F*, *t*, *r*) with confidence intervals, effect sizes, degrees of freedom and *P* value noted<br>*Give P values as exact values whenever suitable.* |
| ☒ | ☐ | For Bayesian analysis, information on the choice of priors and Markov chain Monte Carlo settings |
| ☒ | ☐ | For hierarchical and complex designs, identification of the appropriate level for tests and full reporting of outcomes |
| ☒ | ☐ | Estimates of effect sizes (e.g. Cohen's *d*, Pearson's *r*), indicating how they were calculated |

*Our web collection on statistics for biologists contains articles on many of the points above.*

## Software and code

Policy information about availability of computer code

Data collection

In vivo bioluminescence imaging: Living Image software (PerkinElmer, version 4.5.5) was used for analysis. Regions of interest (ROIs) were defined around the electroporated regions to quantify IVIS signal intensity as photons/second.

Flow cytometry: All samples were measured on a LSR Fortessa (BD Bioscience), compensated on the cytometer using single-stain controls or beads (Miltenyi Biotec, 130-104-693; and Biolegend, 424602) and analyzed in FlowJo v10 (BD Life Science).

Immunohistochemistry: Slides were scanned using the Aperio AT2 slide scanner (Leica, 23AT2100) at 40x magnification. Morphological assessment and quantification of immunohistochemistry and morphological tumor features were conducted with the expertise of pathologist Felix Kommoss in blinded fashion. Digitized image files were analyzed using QuPath software (version 0.4.1) and assembled into final figures using Affinity Designer software (version 1.10.5).

RNAseq: Sequencing was carried out on Illumina’s NovaSeq 6000 S1 or S4 flow cell with paired-end 50 bp reads, yielding an average of approximately 20 million reads per sample. Sequencing reads were aligned to the mouse reference genome (GRCm38mm10) by DKFZ’s Omics IT and Data Management Core Facility (ODCF) using the One Touch Pipeline (OTP)264 / RNAseq workflow pipeline, version 1.3.0 (STAR Version 2.5.3a, Merging/duplication marking program: Sambamba Version 0.6.5. SAMtools program: Version 1.6.), resulting in raw counts, RPKM, and TPM values. K-means analysis was performed using the iDEP web collection of R packages.

DNA Methylation: Genome-wide methylation profiling was performed using the Illumina Infinium Mouse Methylation BeadChip covering > 285,000 CpG sites distributed over the mouse genome. IDAT files were obtained and processed using the R package sesame, version 1.16.1 (SEnsible Step-wise Analysis of DNA MEthylation BeadChips) to generate normalized beta values

Data analysis

RNAseq analysis: Differential gene expression analysis was conducted with DeSeq2 in R Studio, version 2022.07.1. Exploratory data analysis and sample correlation matrix was performed using the iDEP web collection of R packages (Ge et al., 2018). Gene ontology (GO) analysis for genes in k-means cluster was performed using DAVID (https://david.ncifcrf.gov/). For cell deconvolution, RNA Seq data was processed using CibersortX in accordance with the developer's manual (https://cibersortx.stanford.edu) 78. The 'Tabula muris' dataset, a publicly accessible collection of single-cell RNA sequencing data from mice, including >100,000 annotated single cells representing more than 130 cell types across 20 different organs 79 was used to create a reference matrix for cell type annotation. Batch correction parameters were set to S mode (recommended for single cell reference data).

DNA Methylation Analysis: Probe intensities underwent background correction using the p-value with the out-of-band array hybridization approach, followed by a normal-exponential out-of-band approach. Dye bias correction was performed by aligning green and red to the midpoint using the dyeBiasCorrTypeINorm method in sesame. Probes targeting the X and Y chromosomes were excluded. Clustering analysis was performed with R packages Rtsne and umap based on the 2,000 or 10,000 most variably methylated probes, with perplexity values set to 5 for tSNE and 15 for umap clustering. To infer CNV profiles, the method described in R package conumee, version 1.32.0, was adapted for the mouse array. Specifically, a panel of n=60 normal tissue idat files from C57BL6 mice, kindly provided by Marc Zuckermann and Tuyu Zheng (DKFZ) underwent the same sesame correction pipeline, and total probe intensities were quantified across all probes in tumor and normal samples. The background ratio of cancer sample to normal control intensities was determined using the slope of a linear model. Subsequently, the log base 2 ratio of observed vs. expected intensity was calculated for every probe. Probes were binned using sesame according to their mm285 array manifest, utilizing the getBinCoordinates function. For heatmap visualization of copy number variation, the color range representing the log fold change in probe intensities was set to -1 to +1.

Cross-species transcriptome analysis of mouse EPO-GEMMs and human sarcomas: To compare RNA-sequencing profiles between mouse and human sarcoma specimens, data of human sarcomas (TCGA, St. Jude and INFORM), representing common and rare soft-tissue sarcoma entities, was assessed for the top 500 most differentially expressed genes in DEseq2 v1.30.1 (log2FC >2, FDR< 0.05) in each subtype vs all other subtypes. The top 500 most differentially expressed genes were determined in the same way for n=63 mouse samples of primary GEMMs representing 10 subtypes entities. All selected human genes were matched to orthologs in mouse genes. In cases with no orthologs these genes could not be considered in the comparative analysis. Alternatively, all ortholog genes identified and the top 2000 most variable genes were selected. These gene lists were subsequently used to create matrices and batch correction was performed for species, sample type ("Muscle control") and dataset (St. Jude, TCGA and INFORM) using Harmony v0.10 (Korsunsky et al., 2019), implemented in R v4.0.3. These batch corrected values were then subjected to t-SNE clustering, excluding non-relevant entities that could not be clearly assigned to one entity or did not have obvious matching mouse samples (e.g. Osteosarcoma, EwS).

Cross-species methylome analysis of mouse EPO-GEMMs and human sarcomas:To compare methylome profiles between mouse and human specimens, data from 302 human sarcomas from the DKFZ Sarcoma methylation classifier were used, representing 12 distinct sarcoma entities. Comparison of syntenic probes was undertaken, similar to prior analysis de-scribed by Zhou et al. (2022) 48. UCSC liftOver was used to map the Infinium HumanMethyla-tion450 array probe sites from the hg19 to mm10 reference genome. There were 15218 shared syntenic probes identified as those overlapping with MM285k array sites. Beta values from these syntenic probes were computed across human methylomes from the DKFZ Sarcoma clas-sifier and the methylomes of EPO-GEMMs. These were converted to M values. Probes were selected for those significantly associated with at least one tumor subtype (p<0.001) and anti-correlated to species (R2>0.8). Harmony was used to project all samples onto a species-neutral space by correcting for the species as a batching variable. The resulting corrected M value ma-trix was used to generate the reported tSNE figures.

Indel analysis from genomic DNA: sequences were aligned with the wildtype sequences derived from mouse tail genomic DNA, using the TIDE algorithm (Tracking of Indels by Decomposition) (Brinkman et al., 2014) (http://shinyapps.datacurators.nl/tide/) to calculate the percentage of insertions and deletions.

Data was plotted using R v4.0.3 and Graphpad Prism version 8.4.3. Affinity Designer version 1.10.5. and Biorender.com were used to generate graphical illustrations and arrange panels into final figures.

For manuscripts utilizing custom algorithms or software that are central to the research but not yet described in published literature, software must be made available to editors and reviewers. We strongly encourage code deposition in a community repository (e.g. GitHub). See the Nature Portfolio guidelines for submitting code & software for further information.

## Data

Policy information about availability of data

All manuscripts must include a data availability statement. This statement should provide the following information, where applicable:

- Accession codes, unique identifiers, or web links for publicly available datasets
- A description of any restrictions on data availability
- For clinical datasets or third party data, please ensure that the statement adheres to our policy

Gene expression and DNA methylation data acquired for this study are available at the Gene expression omnibus (GEO accession: GSE265875).

## Research involving human participants, their data, or biological material

Policy information about studies with human participants or human data. See also policy information about sex, gender (identity/presentation), and sexual orientation and race, ethnicity and racism.

Reporting on sex and gender: not applicable

Reporting on race, ethnicity, or: not applicable

| | |
|---|---|
| other socially relevant groupings | |
| Population characteristics | *Describe the covariate-relevant population characteristics of the human research participants (e.g. age, genotypic information, past and current diagnosis and treatment categories). If you filled out the behavioural & social sciences study design questions and have nothing to add here, write "See above."* |
| Recruitment | *Describe how participants were recruited. Outline any potential self-selection bias or other biases that may be present and how these are likely to impact results.* |
| Ethics oversight | *Identify the organization(s) that approved the study protocol.* |

Note that full information on the approval of the study protocol must also be provided in the manuscript.

# Field-specific reporting

Please select the one below that is the best fit for your research. If you are not sure, read the appropriate sections before making your selection.

☒ Life sciences ☐ Behavioural & social sciences ☐ Ecological, evolutionary & environmental sciences

For a reference copy of the document with all sections, see nature.com/documents/nr-reporting-summary-flat.pdf

# Life sciences study design

All studies must disclose on these points even when the disclosure is negative.

| | |
|---|---|
| Sample size | Group sizes for in vivo experiments were determined through statistical consultation in the Department of Biostatistics at the DKFZ, which included in silico simulations. |
| Data exclusions | No data exclusions was performed. |
| Replication | All experiments were repeated independently. The number of biological replicates are indicated in the figure legends. |
| Randomization | Group allocation for preclinical treatment studies was determined through random distribution. Tumor-bearing mice were randomized for NTRK-inhibitor treatment in vivo. |
| Blinding | Morphological assessment and quantification of immunohistochemistry and morphological tumor features were conducted with the expertise of pathologist Felix Kommoss in blinded fashion. Outcome assessment for preclinical treatment trials was performed in blinded fashion. Other group allocations and outcome assessments were performed in non-blinded fashion. |

# Reporting for specific materials, systems and methods

We require information from authors about some types of materials, experimental systems and methods used in many studies. Here, indicate whether each material, system or method listed is relevant to your study. If you are not sure if a list item applies to your research, read the appropriate section before selecting a response.

## Materials & experimental systems

| n/a | Involved in the study | |
|---|---|---|
| ☐ | ☒ | Antibodies |
| ☐ | ☒ | Eukaryotic cell lines |
| ☒ | ☐ | Palaeontology and archaeology |
| ☐ | ☒ | Animals and other organisms |
| ☒ | ☐ | Clinical data |
| ☒ | ☐ | Dual use research of concern |
| ☒ | ☐ | Plants |

## Methods

| n/a | Involved in the study | |
|---|---|---|
| ☒ | ☐ | ChIP-seq |
| ☐ | ☒ | Flow cytometry |
| ☒ | ☐ | MRI-based neuroimaging |

## Antibodies

| | |
|---|---|
| Antibodies used | CCas3, IHC, 1:400, Cell Signalling, 9661<br>MyoD1, IHC, 1:100, Abcam, 203383<br>Myogenin, IHC, 1:100, Abcam, 124800<br>Desmin, IHC, 1:300, GeneTex, GTX15200<br>AE1/AE3, IHC, 1:50, DAKO Agilent, M3515<br>CD3, IHC, 1:50, Dianova, DIA303<br>CD45, IHC, 1:100, BD Pharm, 550286<br>HA tag, IHC, 1:800, Cell Signalling, C29F4<br>Ki-67, IHC, 1:100, Abcam, 15580 |

pH2AX, IHC, 1:500, Cell Signalling, 9718
S100, IHC, 1:600, DAKO Agilent
ASMA, IHC, 1:200, Sigma-Aldrich, A2547
GFP, IHC, 1:100, Cell Signalling, 2956
Anti-Goat, IHC, 1:500, Dianova, RbxGT-004-DBIO,
Anti-Mouse, IHC, 1:1000, Dianova, 115-065-062
Anti-Rabbit, IHC, 1:800, Dianova, 111-065-144
Anti-Rat, IHC, 1:200 Vector BA-4001
CD105, Serial IF, 1:40, Miltenyi Biotec, 130-102-915
CD138, Serial IF, 1:50, Miltenyi Biotec, 130-122-945
CD169, Serial IF, 1:50, Miltenyi Biotec, 130-125-523
CD2, Serial IF, 1:40, Miltenyi Biotec, 130-115-958
CD276, Serial IF, 1:50, Santa Cruz Biotechnology, sc-376769PE
CD45, Serial IF, 1:50, Miltenyi Biotec, 130-116-500
CD3, Serial IF, 1:50, Miltenyi Biotec, 130-119-798
CD68, Serial IF, 1:50, Miltenyi Biotec, 130-112-855
CD79b, Serial IF, 1:50, Miltenyi Biotec, 130-105-893
CD90.2, Serial IF, 1:50, Miltenyi Biotec, 130-120-897
Collagen IV, Serial IF, 1:200, Abcam, AB309503
Desmin, Serial IF, 1:50, Santa Cruz Biotechnology, sc-23879PE
H2AX pS139, Serial IF, 1:50, Miltenyi Biotec, 130-125-883
Ki-67, Serial IF, 1:50, Miltenyi Biotec, 130-117-691
Ly-6C, Serial IF, 1:50, Miltenyi Biotec, 130-111-915
Ly-6G, Serial IF, 1:50, Miltenyi Biotec, 130-120-820
MHC Class II, Serial IF, 1:50, Miltenyi Biotec, 130-112-386
Smooth muscle actin 1, Serial IF, 1:50, Miltenyi Biotec, 130-123-363
Smooth muscle actin 2, Serial IF, 1:50, Thermo Fisher Scientific, 53-9760-82
Actin (H+L), WB, 1:500, Sigma, A3854
GFP, D5.1, WB, 1:1000, Abcam, 2956
Luciferase, WB, 1:1000, Promega, G745A
Pan-RAS, WB, 1:1000, BD Pharm, 610001
Anti-Goat IgG (H+L), WB, 1:10.000, Sigma-Aldrich, A8919
Anti-Mouse IgG (H+L),WB, 1:10.000, Life Tech, A16162
Anti-Rabitt IgG (H+L), WB, 1:10.000, Sigma-Aldrich, NA934

Validation

All antibodies were validated by the manufacturers. GFP and HA Tag antibodies (HA, V5) have been extensively used in the literature. Antibodies used for IHC have also been extensively used in the literature. IHC and IF stainings using positive and negative controls were performed when possible

## Eukaryotic cell lines

Policy information about cell lines and Sex and Gender in Research

Cell line source(s)

Human Embryonic Kidney HEK293T cells (RRID:CVCL 0063), human alveolar RMS cell line Rh30 (RRID:CVCL_0041) and murine neuroblastoma cells N2A (CCL-131) were purchased from the American Type Culture Collection (ATCC) and maintained in DMEM (Gibco) supplemented with 10% Fetal Bovine Serum and 1% Penicillin/Streptomycin (P/S). IMT_NTRK1/INF_R_153 carrying ETV6::NTRK3 was generated from a primary tumor biopsy obtained from an inflammatory myofibroblastic tumor (IMT) enrolled in the INFORM registry study and cultured in RPMI (Gibco) + 10% FCS + 1% MEM (minimal essential amino acids) + 1% P/S.

All other cell lines used were derived in these sutdy from tumor cell purification of mouse sarcoma EPO-GEMMs.

Authentication

Human cell lines were authenticated via classical STR profiling with the company Multiplexion and by western blot for oncofusion detection. HEK293-T were obtained authenticated by the manufacturer (ATCC) and by morphology.

Mouse cells lines derived from sarcoma EPO-GEMMs were genotyped for the presence of specifc oncogenes and analysed by TIDE for the presence of indels for specific tumor suppressor genes.

Mycoplasma contamination

All cell lines are monthly tested for mycoplasma and remained negative.

Commonly misidentified lines
(See ICLAC register)

Not applicable

## Animals and other research organisms

Policy information about studies involving animals; ARRIVE guidelines recommended for reporting animal research, and Sex and Gender in Research

Laboratory animals

Mouse strains (CD-1 and C57BL6/J) were purchased from Janvier laboratories and housed at the central DKFZ animal facility under

| | |
|---|---|
| | Specific Opportunist Pathogen-Free (SOPF) conditions, utilizing individually ventilated cages. Animals had ad libitum access to food and water. Daily assessments of their well-being were carried out by certified animal caretakers. |
| Wild animals | not applicable |
| Reporting on sex | Electroporation cohorts always included at least two genetic groups per litter, and male and fe-male mice were included equally in all analyses. Each genotype combination was tested in at least 6 animals (3 males, 3 females). For NTRKi or CART cell treatment onlyt female mice were used. |
| Field-collected samples | not applicable |
| Ethics oversight | All animal experiments conducted in this study were carefully planned and approved by the by the local veterinary authorities and the Regierungspräsidium Karlsruhe, Baden-Wurttemberg, Germany (animal permits G-36/19, G-2/20, G-3/20). The study adhered to the ARRIVE guidelines, European Community and GV-SOLAS recommendations (86/609/EEC), and United Kingdom Coordinating Committee on Cancer Research (UKCCCR) guidelines for the welfare and use of animals in cancer research. Conscientious application of the 3R guideline (replacement, reduction, refinement) was emphasized, prioritizing the reduction of potential suffering for the animals. |

Note that full information on the approval of the study protocol must also be provided in the manuscript.

## Plants

| | |
|---|---|
| Seed stocks | *Report on the source of all seed stocks or other plant material used. If applicable, state the seed stock centre and catalogue number. If plant specimens were collected from the field, describe the collection location, date and sampling procedures.* |
| Novel plant genotypes | *Describe the methods by which all novel plant genotypes were produced. This includes those generated by transgenic approaches, gene editing, chemical/radiation-based mutagenesis and hybridization. For transgenic lines, describe the transformation method, the number of independent lines analyzed and the generation upon which experiments were performed. For gene-edited lines, describe the editor used, the endogenous sequence targeted for editing, the targeting guide RNA sequence (if applicable) and how the editor was applied.* |
| Authentication | *Describe any authentication procedures for each seed stock used or novel genotype generated. Describe any experiments used to assess the effect of a mutation and, where applicable, how potential secondary effects (e.g. second site T-DNA insertions, mosiacism, off-target gene editing) were examined.* |

## Flow Cytometry

### Plots

Confirm that:

- [ ] The axis labels state the marker and fluorochrome used (e.g. CD4-FITC).
- [ ] The axis scales are clearly visible. Include numbers along axes only for bottom left plot of group (a 'group' is an analysis of identical markers).
- [ ] All plots are contour plots with outliers or pseudocolor plots.
- [ ] A numerical value for number of cells or percentage (with statistics) is provided.

### Methodology

| | |
|---|---|
| Sample preparation | Describe the sample preparation, detailing the biological source of the cells and any tissue processing steps used. |
| Instrument | *Identify the instrument used for data collection, specifying make and model number.* |
| Software | Describe the software used to collect and analyze the flow cytometry data. For custom code that has been deposited into a community repository, provide accession details. |
| Cell population abundance | *Describe the abundance of the relevant cell populations within post-sort fractions, providing details on the purity of the samples and how it was determined.* |
| Gating strategy | *Describe the gating strategy used for all relevant experiments, specifying the preliminary FSC/SSC gates of the starting cell population, indicating where boundaries between "positive" and "negative" staining cell populations are defined.* |

- [ ] Tick this box to confirm that a figure exemplifying the gating strategy is provided in the Supplementary Information.
